# On Bronchitis

**Published:** 1852-08

**Authors:** R. B. Todd

**Affiliations:** Physician to King’s College Hospital


From Med. Gazette, Nov. 21, 1851, p. 874.

*On Bronchitis.* By Dr. R. B. Todd, F.R.S., Physician to King's College Hospital.

In a first attack of bronchitis, the lungs are not altered in structure, or the chest in form; but after repeated attacks both are affected in a manner which I shall presently describe to you.

It is remarkable that in these cases we always find that the expiratory sound is more or less prolonged; in some instances a long and somewhat sharp wheeze forms the sound of expiration, which at times is as long and even longer than the inspiratory sound.

In the normal state of respiration you know that the chief sound is that caused by the ingress of air into the lungs, or the inspiratory sound, and that the expiratory sound is so slight that it is scarcely appreciable, and, indeed, in most instances, is inaudible. This is doubtless owing chiefly to the extreme rapidity with which the lung, by its elastic reaction, expels the air; and partly, perhaps, it may be due to the great softness of the pulmonary tissue, which makes it an imperfect conductor of sound. When, however, the bronchial membrane is thickened by inflammation, and the tubes narrowed by the same cause, and their canals impeded by mucus, the air passes out of the lung with difficulty, in consequence of the obstacle to its exit which it thus meets with. Moreover, the pulmonary tissue around the tubes is increased in density, and becomes, in virtue of that increase, a better conductor of sound, so that not only is the exit of the air from the lung much retarded, but its outward passage is rendered much more audible.

Such are the changes and physical signs which we find in the early attacks of bronchitis. But when there have been repeated attacks of this disease—and especially where these acute attacks have supervened upon a continuous chronic bronchitic state (if I may be allowed to coin such an adjective)—the lungs undergo very serious changes, which interfere greatly with their functional integrity and activity. These changes are of various kinds: sometimes one or more bronchial tubes are obliterated (as first, I believe pointed out by Dr. Stokes), and the pulmonary lobule or lobules to which they lead collapse from the absence of their usual distending medium, the air, and become more or less wasted. The adjacent tubes and their corresponding air-cells dilate to receive more air, just as the tubes of one lung would if the other were compressed by fluid, but probably to a much greater degree; and thus we may have in the same lung at parts a collapse and an atrophy of portions of the lung, and at other points expansion and permanent dilatation of the air-tubes and air-cells.

But there are other and more potent causes in operation to promote the dilatation of the air-cells and tubes. These are the disturbed state of breathing caused by the bronchial irritation, and more especially the difficulty of expiration, and the mischief done to the tissues of the bronchial apparatus by the repeated attacks of inflammation. Thus the bronchial irritation gives rise to a more or less asthmatic state, in which the inspiration is performed with considerable force, and that in a state of lung which is ill-suited to resist the pressure of the in-rushing air. The muscular fibres of the bronchial tubes must, by the repeated attacks of inflammation of the mucous membrane, be more or less weakened. Now, the most probable office of these fibres is to regulate the admission of air into the lung, and thereby to protect its delicate tissues against undue pressure, just as the muscular fibres of the arteries regulate the flow of blood into them, and to a certain extent antagonise the heart's force. Hence in an enfeebled state of this muscular apparatus the bronchial tubes will yield under the force of the inspired air, and become more or less dilated; and an undue quantity of air will rush in most abundantly at those parts where the muscles are weakest, and therefore afford least resistance.

Again, when the air has accumulated in the lung, it is with difficulty expelled. There are direct obstacles to its outward passage in the altered condition of the mucous membrane of the tubes, and in the accumulation of secretion in them. Moreover, the expelling force is in great part due to the reaction of the elastic tissue, which enters largely into the formation of bronchial tubes, and which invests the lobules of the lung. But the undue stretching to which this tissue has been subjected, not only from the forced inspiration, but from the detention of the air within the lungs, and the accumulation of mucous in the tubes, must, as the disease advances, more or less impair its elastic power, and therefore weaken the force which takes the most direct and the largest share in the process of expiration. Thus the longer the duration of the disease, and the more frequent the attacks, the more serious will be the evils which follow in its train.

You may readily gather from what I have already said what are the alterations in the lung which chronic bronchitis tends to produce. They are—first, the immediate changes, and, secondly, the remote ones. The immediate changes are those which affect the mucous membrane and muscular fibres of the bronchial tubes, as well as the tubes themselves; such as inflammation, thickening, altered secretions—perhaps even ulceration—and also more or less dilatation of the tubes. The remote changes are a still further dilatation of the tubes—a dilatation of the air-cells; and when that dilatation goes beyond a certain point, a stretching, and even a rupture of many of the bands of elastic tissue which are found in the lobules. This stretching of the bronchial passages and cells gives rise to a corresponding change in the air-cells, which exercises a very marked influence upon the capillary vessels of the lung, which, so far as I know, was pointed out by Mr. Rainey, of St. Thomas's Hospital. The expansion of the air-cells causes an extension of the meshes of the capillary net-work distributed upon and within them; and the rupture of many of the intersecting bands of fibrous tissue causes obliteration of their blood vessels. Thus the capillary system of the lung becomes diminished in its capacity, and thus is explained the fact long known, that emphysematous lungs are apt to be pale, and to look as if they contained but little blood.

Now, the state to which the lung is thus brought by a long continuance of chronic bronchitis, is that which we call *emphysema*, in which there is more or less dilatation of a greater or less number of air-cells, and a consequent diminution in the area of the capillary system belonging to them.

Chronic bronchitis, however, is not the sole cause of emphysema, although certainly the most frequent. That state of lung will follow repeated attacks of asthma; and it may be caused by great and prolonged efforts; and there are those who believe that it may arise even in the absence of any such exciting causes, in persons who have a certain constitutional weakness of the lungs, which may be inherited.

In both the acute and chronic forms of bronchitis one of the most valuable remedies is counter-irritation. This I employ very freely in these cases, not so much by blisters as by turpentine or mustard, and there is this great advantage in this mode of counter-irritation, that you can apply it frequently and at short intervals, and moreover it is immediate in its effects, whereas a blister takes several hours to produce vesication, and it cannot be speedily re-applied. Dry cupping is also a useful form of counter-irritation, and very applicable to such cases as I have mentioned.

Generally speaking, patients laboring under bronchitis, and especially those who have had many attacks, are not very tolerant of a depleting or depressing treatment. General bleeding by venesection is, in many instances, highly dangerous; topical bleeding is borne better; when tried, only a small quantity of blood should be taken.

The medicines most applicable to these cases are those which produce a free diaphoresis, and expectorants, sometimes sedatives. When the expectoration is viscid, and sticks to the tubes so as to make it difficult of dislodgment, great benefit often results from the cautious use of tartarized antimony in small doses; but this must be used only for a very short time, as it tends to produce a profuse watery expectoration, and very much to weaken the patient; as soon, therefore, as the very viscid character of the expectoration is overcome, it should be given up.

When you wish to promote expectoration without causing any undue increase in the quantity of secretion, you will find nothing better than ammonia. In bronchial catarrhs, if there be fever, it may be given freely with the liquor ammoniæ acetatis, and you thus get a copious diaphoresis also. I am also in the habit of using the chloric ether pretty freely in bronchial attacks, either alone or conjoined with ammonia. It is a valuable stimulating expectorant, and has some sedative influence likewise: if not given in too large a dose it is an agreeable medicine to take, and forms a pleasant ingredient for a cough mixture. The decoction of the polygala senega is much lauded for its influence on bronchial affections; I have given it very freely, and, except for its unpleasant taste, can find no fault with it, nor can I bestow upon it any very strong encomiums.

With the use of sedatives you require caution, especially with opium. Conium, hyoscyamus, hop, &c., are well borne on the whole; but nothing relieves irritable cough so effectually as opium; yet when there is much bronchial congestion, you will beware of using it too freely, as it unquestionably tends to increase that, and to endanger the life of the patient. On the other hand, when expectoration is free or too profuse, a moderate dose of opium often exercises the most beneficial influence, procures sleep, moderates expectoration, and relieves the cough. The reputation of the old paregoric elixir, modernised into compound tincture of camphor, is likely to last even through these days of scepticism.

In the more advanced stages, and especially if there be sweats, tonics are useful, and sometimes astringents containing tannin, or even the tannic or gallic acid.

